# Concurrent Acute Ischemic Stroke and Non-Aneurysmal Subarachnoid Hemorrhage in COVID-19

**DOI:** 10.1017/cjn.2020.242

**Published:** 2020-11-05

**Authors:** Lennie Lynn C. de Castillo, Jose Danilo B. Diestro, Katrina Hannah D. Ignacio, Karl Josef Niño J. Separa, Paul Matthew D. Pasco, Maria Carissa P. Franks

**Affiliations:** Department of Neurosciences, College of Medicine and Philippine General Hospital, University of the Philippines Manila, Manila, Philippines; Département de radiologie, radio-oncologie et médecine nucléaire, Centre Hospitalier de l’Université de Montréal, Université de Montréal, Montréal, Canada

**Keywords:** COVID-19, Acute Ischemic Stroke, Subarachnoid Hemorrhage

Cerebrovascular disease has been described as a potential sequela of the novel coronavirus disease of 2019 (COVID-19) with one study reporting its occurrence in 106 patients. In this rapid review, 83% of the patients had ischemic strokes, while 17% were hemorrhagic.^1^ Most of the hemorrhagic strokes were either intracerebral hemorrhage or aneurysmal subarachnoid hemorrhage. The mortality of COVID-19 patients with cerebrovascular events can be as high as 38%.^2^ As of this writing, there has been no reported case of a COVID-19 patient with concurrent acute ischemic and hemorrhagic strokes.

**Figure 1:**
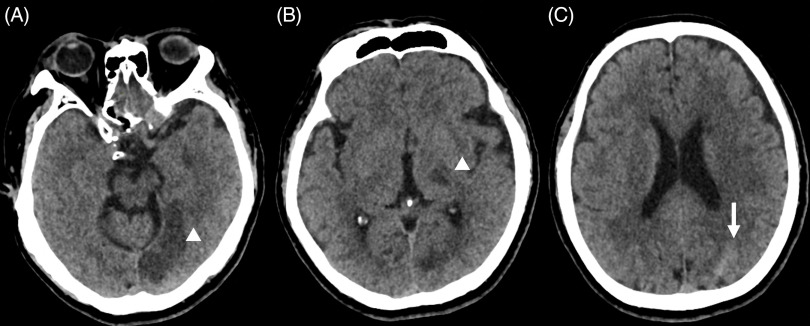
Plain cranial CT scan showing. (A) Left temporal lobe infarct (white arrowhead). (B) Left thalamic infarct (white arrowhead). (C) Subarachnoid hemorrhage at the left posterior parietal area (white arrow).

We report a case of a 64-year-old male, hypertensive and smoker, who came to our emergency department for sudden onset right-sided weakness, numbness, and dysarthria. There was no history of trauma or respiratory symptoms prior to admission. He had no prior use of antithrombotics. He works as a utility worker in the designated COVID-19 area of the hospital. On examination, he had a blood pressure of 150/90 with a normal heart rate and regular rhythm right superior quadrantanopia, right hypesthesia, and right hemiparesis. His National Institute of Health Stroke Scale (NIHSS) score was 7. A 12-lead electrocardiogram showed sinus rhythm. A plain cranial CT scan revealed an acute infarct on the left thalamus and left temporo-occipital lobe, as well as a small subarachnoid hemorrhage on the left parietal sulcus (Figure 1). The cranial CT angiogram (CTA) did not show any acute large vessel occlusions, aneurysms, or venous thrombosis. His severe acute respiratory syndrome coronavirus-2 reverse transcriptase polymerase chain reaction (SARS-CoV 2 RT-PCR) was positive. Chest X-ray showed pneumonia. He had a normal complete blood count, prothrombin time, international normalized ratio, partial thromboplastin time, ferritin, erythrocyte sedimentation rate, and high sensitivity C-reactive protein. D-Dimer and lactate dehydrogenase were slightly elevated at 0.66 mcg/ml and 209 U/l, respectively. Institutional limitations precluded an urgent catheter angiogram. He was started on Aspirin 160 mg once a day for secondary stroke prevention after an aneurysm was ruled out and was discharged with a modified Rankin scale score of 4 after 14 days. Anticoagulation was not given due to the concurrent subarachnoid hemorrhage.

Recent studies reported that COVID-19 increases the risk of cerebrovascular diseases. The exact mechanism by which the virus can involve the nervous system is still unclear. However, the proposed pathophysiology suggests that it may be a multifactorial process. Possible routes of SARS-CoV-2 in the cerebrovascular system include the olfactory epithelium and the respiratory tract with subsequent viremia.^1^ The virus can bind to the ACE-2 receptors of the cerebrovascular endothelium and causes endothelial damage and induces cytokine storm, which can then lead to vasculitis and subsequent stroke.^3^ Endothelial dysfunction and the consequent hyperinflammatory reaction may also increase the risk of plaque rupture and thrombosis.^4^ A hypercoagulable state is also another mechanism by which the virus can cause stroke, as evidenced by the increase in D-dimer seen in our patient.^5,6^ These mechanisms may explain why our patient had ischemic stroke. The disease is also associated with a downregulation of the ACE-2 receptors, elevated angiotensin II levels, and aforementioned endothelial damage, which can cause an increase in blood pressure and subsequently increase the risk for hemorrhagic stroke.^4^ The presence of other well-known risk factors for stroke, such as hypertension and smoking in our patient may have placed him at a greater risk for acquiring COVID-19 and developing cerebrovascular disease.

Non-aneurysmal SAH has previously been reported in a COVID-19 patient.^7^ However, the co-occurrence of acute ischemic stroke and non-aneurysmal SAH has not been documented previously. The location of the patient’s subarachnoid hemorrhage is far from the infarcted tissue, making the diagnosis of a hemorrhagic infarct or a delayed cerebral ischemia from vasospasm unlikely. This suggests the possibility that the proposed mechanisms in COVID-19-related stroke can happen simultaneously, predisposing the patients to both types of stroke. Whether this finding is associated with COVID-19 or just coincidental is yet to be elucidated in future researches.
